# Intracellular Penetration of Atazanavir, Ritonavir and Dolutegravir With Concomitant Rifampicin: A Dose Escalation Study

**DOI:** 10.1002/cpt.3572

**Published:** 2025-01-31

**Authors:** Amedeo De Nicolò, Alice Palermiti, Henry Mugerwa, Shamim Nakabuye, Josephine Namusanje, Josephine Kobusingye, Denis Odoch, Mohammed Lamorde, Allan Kengo, Paolo Denti, Kamunkhwala Gausi, Gary Maartens, Helen McIlleron, Lubbe Wiesner, Saye Khoo, Catriona Waitt, Antonio D'Avolio

**Affiliations:** ^1^ Laboratory of Clinical Pharmacology and Pharmacogenetics, Department of Medical Sciences University of Turin Turin Italy; ^2^ Joint Clinical Research Centre Kampala Uganda; ^3^ Infectious Diseases Institute Makerere University College of Health Sciences Kampala Uganda; ^4^ Division of Clinical Pharmacology, Department of Medicine University of Cape Town Cape Town South Africa; ^5^ Department of Pharmacology and Therapeutics University of Liverpool Liverpool UK

## Abstract

Ritonavir‐boosted atazanavir is a victim of drug–drug interaction with rifampicin, a key component of antitubercular treatment. In a recent dose escalation clinical trial, we showed that increasing atazanavir/ritonavir to 300/100 mg b.i.d. compensates for reduced drug exposure in plasma due to rifampicin, but the intracellular effects remained unexplored. This sub‐study investigated the intracellular penetration of atazanavir/ritonavir and dolutegravir into peripheral blood mononuclear cells (PBMC). Twenty‐six healthy volunteers living with HIV, virologically suppressed, and taking atazanavir/ritonavir containing regimens were enrolled. The trial consisted of four sequential periods: PK1, participants were on atazanavir/ritonavir 300/100 mg q.d.; at PK2, rifampicin 600 mg q.d. and dolutegravir 50 mg b.i.d. were added (2 weeks); at PK3, atazanavir/ritonavir dose was increased to 300/100 mg b.i.d. (1 week); at PK4, rifampicin dose was doubled (1 week). Atazanavir, ritonavir, and dolutegravir were quantified in plasma and PBMC using LC–MS/MS methods to evaluate steady‐state concentrations at the end of each period. Atazanavir/ritonavir dose escalation successfully restored intracellular concentrations comparable to those observed without rifampicin, with a geometric mean ratio of 0.99 (CI_90_ 0.72–1.41) for atazanavir at PK3 compared with PK1. The intracellular concentration of dolutegravir increased significantly with atazanavir/ritonavir dose escalation, similar to plasma. Finally, further, increasing the rifampicin dose did not show an additional impact on atazanavir/ritonavir concentrations in PBMC. The study confirms that increasing the ATV/r dose can be an effective strategy for compensating rifampicin effects even at the intracellular level, supporting its use in clinical settings.

**Study Highlights**

**WHAT IS THE CURRENT KNOWLEDGE ON THE TOPIC?**

Ritonavir‐boosted atazanavir (ATV/r) is an important anti‐HIV therapeutic choice which is, however, victim of interaction with rifampicin. It was shown that increasing ATV/r dose to twice daily may overcome this pharmacokinetic interaction, considering their concentrations in plasma.

**WHAT QUESTION DID THIS STUDY ADDRESS?**

Despite the dose escalation of ATV/r is known to be capable of restore drug levels in plasma in the presence of rifampicin, it was not known if this is valid also within the target cells, PBMCs. This is important since rifampicin induces drug efflux transporters so that the intracellular penetration relative to the plasma could change, potentially leading to suboptimal penetration in the target cells even in the presence of adequate concentrations in plasma.

**WHAT DOES THIS STUDY ADD TO OUR KNOWLEDGE?**

The results from this study confirmed that the dose escalation strategy is capable of restoring ATV/r concentrations within the intracellular compartment comparable to those observed before the introduction of rifampicin, providing an important validation to the evidence from the plasma compartment, with pharmacodynamic and clinical implications.

**HOW MIGHT THIS CHANGE CLINICAL PHARMACOLOGY OR TRANSLATIONAL SCIENCE?**

This study may change the clinical practice in the management of the HIV‐tuberculosis coinfection, particularly in sub‐Saharan Africa, since it demonstrates ATV/r‐based regimens can be continued with a simple change from once to twice daily schedule in case of treatment with RIF, with considerable confidence about the theoretical effectiveness and safety of this approach. On the other hand, the evidence of inter‐ and intra‐individual variability in the intracellular penetration of the antiretroviral drugs suggests that the intracellular evaluation should be considered useful in future DDI studies.

The combination of a backbone of nucleoside reverse transcriptase inhibitors (NRTIs) with a protease inhibitor (PI) boosted with ritonavir (RTV; bPI) is recommended as second‐line treatment for HIV by WHO.^1^ Among PIs, a WHO‐preferred choice is boosted atazanavir (ATV/r) due to generally good tolerability and convenient once‐daily dosing.^1^

The incidence of concomitant infection with tuberculosis is particularly high in sub‐Saharan Africa, complicating the clinical management of these cases.^2^, ^3^ A key component of first‐line antitubercular treatment is rifampicin (RIF), which is a strong inducer of many enzymes and transporters, such as CYP3A4 and P‐glycoprotein (P‐gP), involved in drug metabolism, distribution, and elimination: concomitant administration of RIF can reduce the concentrations of drugs that are substrates of these enzymes and transporters, leading to clinically relevant drug–drug interactions (DDIs).^4^ Notably, ATV/r, as well as other bPIs, are substrates, inhibitors, and weak inducers of CYP3A4,^5^, ^6^, ^7^, ^8^, ^9^, ^10^, ^11^, ^12^ so a significant and clinically relevant reduction of their exposure is expected with RIF.^1^, ^13^, ^14^ Moreover, some DDI studies have shown increased toxicity emerging from the association of bPIs and RIF.^15^, ^16^, ^17^, ^18^, ^19^, ^20^

Recently, the impact of this interaction and the capability of a dose increase of ATV/r to mitigate it was predicted by physiologically based pharmacokinetic (PBPK) modeling^7^ and tested in healthy people living with HIV (PLWH) without tuberculosis coinfection in a prospective, open‐label, dose escalation study.^21^ This study showed that ATV/r escalation to 300/100 mg twice daily (b.i.d.) was successful in most participants to overcome the DDI with RIF, even when RIF was administered at the higher dose of 1,200 mg/day. In this trial, dolutegravir (DTG) was also administered at 50 mg b.i.d. as additional protection in case suboptimal concentrations of ATV/r resulted in viral rebound.

The ability of dose escalation to compensate for this DDI, and similar ones, has generally been evaluated on plasma drug concentrations,^15^, ^16^, ^17^, ^19^, ^20^ but it is important to note that the pharmacological targets of most ARVs are inside the cells, in lymphocytes and monocytes/macrophages. Therefore, the intracellular concentration of ARVs was described to better reflect their effect, and in some cases, toxicity.^22^, ^23^, ^24^, ^25^ Moreover, despite being generally higher the concentrations in PBMC are considered more representative and are used as comparative reference for the ones from lymphoid tissues, than the ones in plasma.^24^, ^26^, ^27^, ^28^ To date, there is no evidence of the impact of the DDI between ATV/r and RIF on the relative intracellular penetration of ATV/r, compared with plasma. There is a possibility of a mismatch between the PK in plasma and that within peripheral blood mononuclear cells (PBMC, circulating lymphocytes, and monocytes), because of genetic differences between patients and the effects of concomitant drugs on drug transporters (e.g., inhibition or induction). In particular, the inductive effect of RIF is expected to have some impact on the expression of P‐gP in PBMC,^29^ potentially reducing the relative penetration of ATV and RTV in this compartment, as reported for other P‐gP substrates.^12^, ^30^, ^31^, ^32^ This could cause clinically significant mismatches between the evidence of the DDI studies in plasma and in PBMC, since differential expression/activity of transporters could yield insufficient intracellular exposure even with sufficient concentrations in plasma.

Therefore, we aimed to describe the intracellular penetration of ATV, RTV, and DTG during the DDI with RIF and the capability of dose escalation to compensate for this DDI within PBMCs, potentially validating the results from the analysis in plasma.

## METHODS

### Study design and participants

Healthy adult PLWH who gave written informed consent, without significant coinfections and on stable treatment with a backbone of two NRTIs and ATV/r for at least 6 months and with undetectable viral load (<50 copies/ml) were enrolled in the DERIVE study (NCT04121195, funded by EDCTP). Exclusion criteria included active tuberculosis (controlled by symptom checklist, examination, and chest X‐ray), active hepatitis B or the use of potentially interacting concomitant drugs, and in female participants, the state of pregnancy, breastfeeding, or the lack of effective contraception.

Ethical approvals were obtained by the Ethics Committee of the Joint Clinical Research Centre (JCRC) of Kampala, Uganda, as well as from the Ugandan National Council for Science and Technology and the Uganda National Drug Authority.

DERIVE was a prospective, open‐label dose escalation study for ATV/r, maintaining a standard dose of the NRTI backbone, consisting of five sequential treatment periods (depicted in **Table** **1**): in period 1, patients maintained the standard 300/100 mg q.d. ATV/r dose for 1 week; in period 2 (2 weeks), RIF 600 mg/day and DTG 50 mg bid were added, to evaluate the enzyme induction without significant risk of virological failure; in period 3 (1 week), the ATV/r dose was increased to 300/100 mg b.i.d., as for the optimal dose predicted by PBPK modeling^7^; in period 4, the RIF dose was further increased to the maximum dose of 1,200 mg q.d., to evaluate potential increased enzyme induction (1 week), and finally, in period 5 RIF was stopped, ATV/r dose was reduced to 300/100 mg q.d., while DTG was kept for further 2 weeks, to allow enzyme induction to wane. The safety follow‐up continued for up to 60 days and then patients exited the study.

**Table 1:** Summary depiction of the study design, treatment periods, and PK evaluations

Period	Days	Study drug (mg)	2 NRTIs	DTG	RIF	Safety (blood chemistry)	PK evaluation	
							Plasma	PBMC
1	1–7	ATV/r 300/100 q.d.	*Standard of Care Doses*	—	—	—	PK1 on day 7	
							Intensive sampling	Trough sampling at 24 hours
2	8–21	ATV/r 300/100 q.d.		50 mg b.i.d.	600 mg q.d.	Days 10, 12, 15, and 18	PK1 on day 7	
							Intensive sampling	Single sampling at 12 hours
3	22–28	ATV/r 300/100 b.i.d.				Days 24 and 26	PK3 on day 28	
							Intensive sampling	Through sampling at 12 hours
4	29–35	ATV/r 300/100 b.i.d.			1,200 mg q.d.	Days 31 and 33	PK4 on day 35	
							Intensive sampling	Trough sampling at 12 hours
5	36–42 *Washout*	ATV/r 300/100 q.d.			—	—	—	—

### PK sampling and PBMC isolation

PK evaluation was conducted at the end of each treatment period, from period 1 to period 4 (PK1 to PK4). For the evaluation of plasma concentrations, sampling of plasma was performed prior to, and at 0.5, 1, 2, 4, 6, 8, and 12 hours relative to directly observed dosing for all the PK visits, with an additional blood sample at 24 hours drawn on PK1, to obtain the trough concentrations for the standard q.d. dose. Considering the need for a larger blood volume and the more complex isolation protocol, blood sampling for PBMC isolation was performed once at each PK visit: at 24 hour post‐dose for PK1 and at 12 hour post‐dose at the following PK visits. On PK2, despite the ATV/r dosing remaining q.d., sampling for PBMC isolation was performed at 12 hours because it corresponded to the real trough concentration for concomitant DTG (given b.i.d.), and to avoid the risk of finding too high a proportion of concentrations under the LLOQ. PBMC were isolated using CPT tubes for density gradient separation to minimize red blood cell contamination. The protocol included two rapid wash steps with 0.9% NaCl at 4°C, as previously described.^19^, ^20^

### PK analysis

The concentrations of ATV, RTV, and DTG in plasma were measured at the University of Cape Town using validated LC–MS/MS methods at each intensive PK timepoint, as described previously.^21^ The LLOQ for the analysis in plasma were 30 ng/ml for ATV and DTG and 5 ng/ml for RTV. Concentrations in PBMC were measured at the University of Turin using a validated UHPLC–MS/MS method as described previously.^24^, ^33^

Then, the observed intracellular amounts of each drug were normalized by the volume of cells in each sample, calculated by multiplying the cell numbers and a mean cell volume of 283 fL, as described previously, obtaining intracellular concentrations expressed in ng/ml.^24^, ^34^ The LLOQ for the intracellular quantification was 0.039 ng/sample for all the drugs, corresponding to an approximate intracellular concentration of nearly 14 ng/ml considering a typical PBMC aliquot of 10 million cells, while the LOD was 0.019 ng/sample (7 ng/ml). Concentrations under the LLOQ were imputed in the statistical analysis as LLOQ/2 (corresponding to the concentration of the LOD, 7 ng/ml), while concentrations under the LOD (7 ng/ml) were imputed as 1 for the sake of log transformation and depicted as 0 in the graphs.

The PBMC/plasma concentration ratios at each visit were calculated by comparing the concentrations at the same time points (24 hour at PK1, 12 hour at all the other PK visits).

### Power calculation and statistical analysis

Power analysis was based on previously published data on ATV, aiming to detect a 20% decrease in the exposure between PK3 and PK1 with 95% power. This resulted in a minimum sample size of 24 patients. As described previously, non‐compartmental analysis (NCA) was applied to the intensive PK data in plasma through the R software (version 4.1) and the ncappc package.^21^

All the PK data were described as geometric means, with 90% confidence intervals (CI90). The longitudinal comparisons between treatment periods were performed by Student's *t*‐test for coupled samples and described through geometric mean and geometric mean ratios (GMR), both with 90% confidence intervals (CI_90_). The PBMC/plasma concentration ratios at each visit were calculated by comparing the concentrations at the same time points (24 hour at PK1, 12 hour at all the other PK visits).

Correlations between intracellular trough concentrations (concentration at 12 hours for PK2) and the plasma PK parameters were tested by Spearman correlation.

## RESULTS

### Patients' baseline characteristics

A comprehensive description of the enrolled volunteers was described in a previous article.^21^ Briefly, 26 participants were enrolled, mainly women (23, 88.4%) with a median (range) age of 44 (28–61) years, and weight of 67 (50–75) kg. They had normal transaminase levels, with median (range) ALT 14 U/L (6.6–33.0) and AST 21.6 (12.4–29.0). The NRTI backbone was based on lamivudine with tenofovir disoproxil fumarate (17 patients), zidovudine (eight patients) or abacavir (one patient).

### Intracellular ATV/r concentrations

Detailed geometric mean and 90% confidence intervals for intracellular trough concentrations and comparisons with concentrations in plasma for each drug are reported in **Table** **2**. A detailed summary of the results for plasma concentrations is provided in supplementary materials. All the samples except a single specimen at PK2 showed detectable ATV concentrations in the PBMC compartment.

**Table 2:** Summary of geometric mean and geometric mean ratios (with 90% confidence intervals) for the trough ARV concentrations in PBMC and plasma throughout the protocol

Drug and matrix	Geometric mean (90% CI)				Geometric mean ratio (90% CI)			
	ATV/r 300/100 q.d. (PK1) C24 hours	ATV/r 300/100 q.d. + RIF 600 (PK2) C12 hours or C24 hours^a^	ATV/r 300/100 b.i.d. + RIF 600 (PK3) C12 hours	ATV/r 300/100 b.i.d. + RIF 1,200 (PK4) C12 hours	PK2 vs. PK1^b^	PK3 vs. PK1	PK4 vs. PK1	PK4 vs. PK3
ATV PBMC *C*_trough_ (ng/ml)	391 (299–510)	65 (37–114)	394 (252–617)	421 (296–597)	0.18 (0.12–0.28)	0.99 (0.72–1.41)	1.08 (0.85–1.37)	1.07 (0.75–1.53)
ATV plasma *C*_trough_ (ng/ml)	590 (480–744)	23 (15–29)	490 (340–700)	480 (360–650)	0.039 (0.032–0.048)	0.83 (0.64–1.1)	0.81 (0.63–1.0)	0.98 (0.81–1.2)
RTV PBMC *C*_trough_ (ng/ml)	113 (77–166)	31 (18–55)	86 (46–148)	99 (63–156)	0.28 (0.18–0.46)	0.73 (0.47–1.13)	0.94 (0.67–1.31)	1.20 (0.80–1.80)
RTV plasma *C*_trough_ (ng/ml)	68 (60–77)	3.8 (3.2–4.5)	38 (29–49)	31 (23–42)	0.056 (0.048–0.065)	0.56 (0.46–0.68)	0.46 (0.35–0.60)	0.83 (0.67–1.00)

						PK3 vs. PK2	PK4 vs. PK2	PK4 vs. PK3
DTG PBMC *C*_trough_ (ng/ml)	—	417 (295–661)	872 (661–1,175)	692 (502–955)	—	1.94 (1.34–2.83)	1.51 (1.07–2.15)	0.78 (0.62–0.98)
DTG plasma *C*_trough_ (ng/ml)	—	1,986 (1,618–2,438)	3,981 (3,365–4,721)	3,828 (3,251–4,498)	—	2.00 (1.70–2.3)	1.90 (1.70–2.20)	0.96 (0.85–1.10)

^a^ Intra‐PBMC concentration at PK2 was evaluated at 12 h, and plasma concentrations at 24 h for ATV and RTV.

^b^ C12 hour (PK2) vs. C24 hour (PK1) comparison for PBMC concentrations.

**Figure 1:**
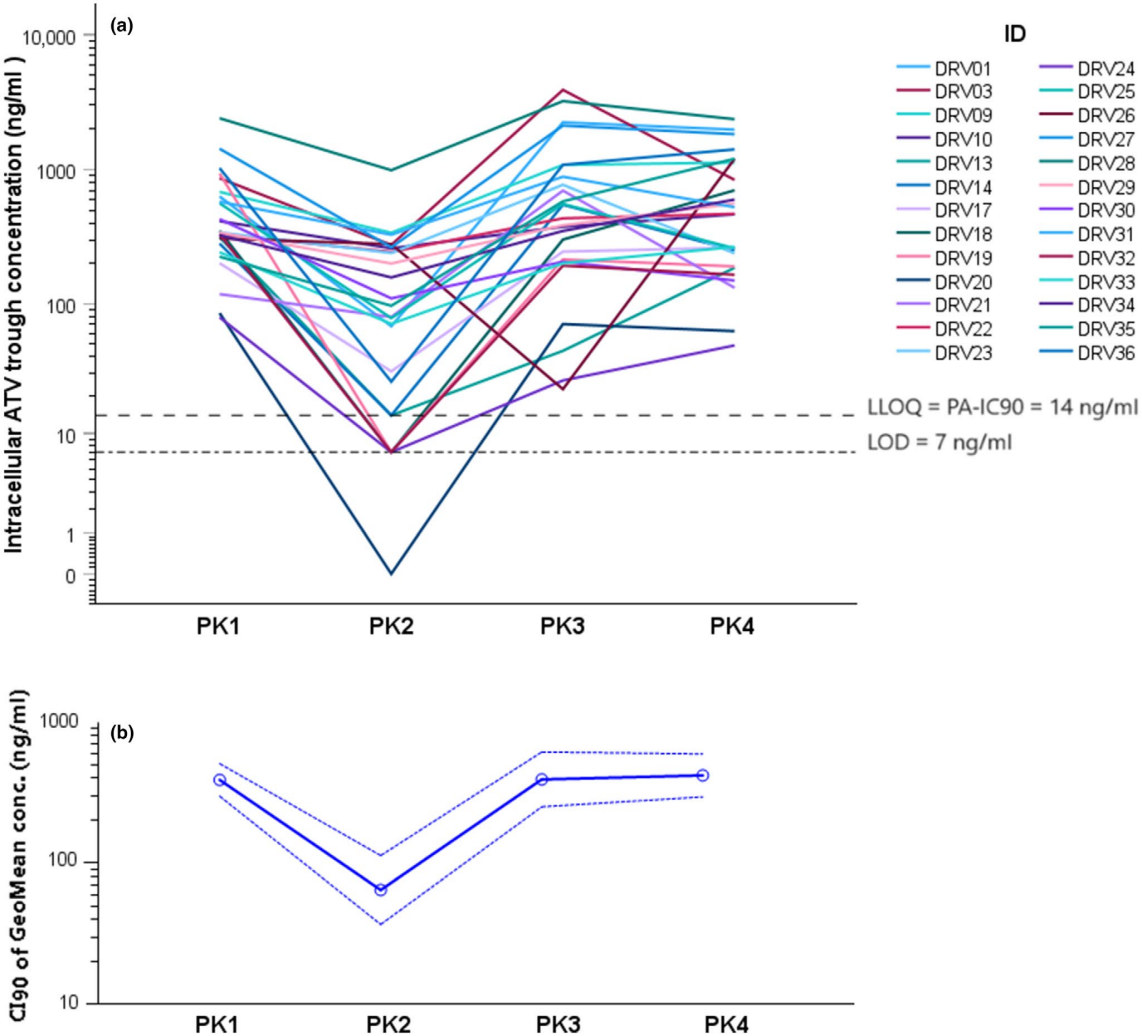
Depiction of the individual trends in intra‐PBMC concentrations of atazanavir (panel **a**) and 90% CI of the geomean concentration (panel **b**) changes throughout the study. Dotted lines represent the boundaries of the CI, solid line is the geomean. PK1 = ATV/r 300/100 mg q.d.; PK2 = addition of RIF 600 mg q.d. and DTG 50 mg b.i.d.; PK3 = ATV/r dose escalation to 300/100 mg b.i.d.; PK4 = RIF dose increased to 1,200 q.d. Concentration at PK2 was determined at 12 hours, despite q.d. dosing.

The intracellular quantification of ATV confirmed a strong reduction in its exposure at C12 hour after the addition of RIF at PK2, with a GMR of 0.18 (0.12–0.28) (**Figure** **1**). ATV/r dose escalation at PK 3 yielded comparable ATV trough concentrations with PK1 in PBMC, with a GMR of 0.99 (0.72–1.41); further increasing RIF dose at PK4 did not yield a significant decrease in the ATV *C*_trough_ concentration in PBMC compared with PK3, with a GMR 1.07 (0.75–1.37).

**Figure 2:**
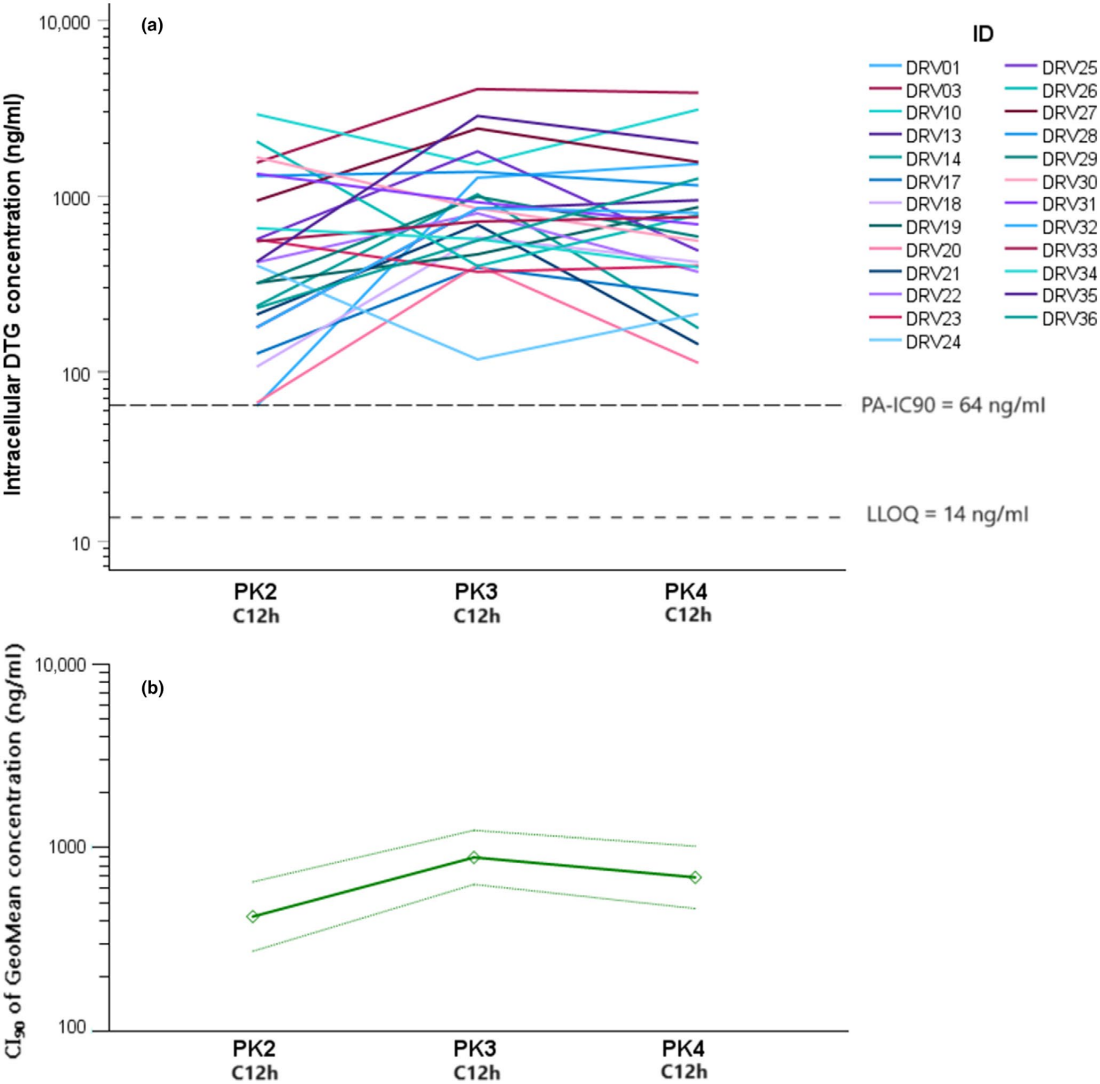
Depiction of the individual trends in intra‐PBMC concentrations of dolutegravir (panel **a**) and 90% CI of the geomean concentration (panel **b**) changes throughout the study. Dotted lines represent the boundaries of the CI, solid line is the geomean. PK2 = addition of RIF 600 mg q.d. and DTG 50 mg b.i.d.; PK3 = ATV/r dose escalation to 300/100 mg b.i.d.; PK4 = RIF dose increased to 1,200 q.d.

PBMC/plasma concentration ratios for ATV (**Table** **3**) changed from 0.65 (0.51–0.84) at PK1 to 0.93 (0.57–1.36) at PK3, after dose escalation, with a GMR 1.33 (0.96–1.84). The RIF dose did not significantly impact the intracellular compartmentalization of ATV, with a GMR of 1.09 (0.77–1.53) on PK4 compared with PK3. All the concentrations of ATV at PK1, PK3, and PK4 in PBMC were above the PA‐IC_90_ value of 14 ng/ml,^35^ which is typically used as minimum target exposure in the extracellular compartment (plasma), as depicted in **Figure** **2**. Conversely, at PK2, four patients showed drug concentrations below the LLOQ (and PA‐IC_90_) at 12 hours post‐dose.

**Table 3:** Summary of geometric mean and geometric mean ratios (with 90% confidence intervals) for the PBMC/plasma trough concentration ratios throughout the protocol

Drug and matrix	Geometric mean (90% CI)				Geometric mean ratio (90% CI)			
	ATV/r 300/100 q.d. (PK1) 24 hour	ATV/r 300/100 q.d. + RIF 600 + DTG 50 b.i.d. (PK2) 12 hour	ATV/r 300/100 b.i.d. + RIF 600 + DTG 50 b.i.d. (PK3) 12 hour	ATV/r 300/100 b.i.d. + RIF 1,200 + DTG 50 b.i.d. (PK4) 12 hour	PK2 vs. PK1	PK3 vs. PK1	PK4 vs. PK1	PK4 vs. PK3
ATV PBMC/plasma ratio	0.65 (0.51–0.84)	0.93 (0.61–1.36)	0.80 (0.57–1.15)	0.88 (0.63–1.22)	1.39 (0.90–2.14)	1.22 (0.91–1.65)	1.33 (0.96–1.84)	1.09 (0.77–1.53)
RTV PBMC/plasma ratio	1.69 (1.20–2.37)	3.24 (2.42–4.42)	2.21 (1.29–3.80)	3.77 (2.69–5.28)	1.66 (1.28–2.15)	1.31 (0.85–2.01)	2.04 (1.39–2.98)	1.43 (1.02–2.02)

						PK3 vs. PK2	PK4 vs. PK2	PK4 vs. PK3
DTG PBMC/plasma ratio	—	0.21 (0.16–0.29)	0.23 (0.18–0.28)	0.18 (0.14–0.25)	—	0.96 (0.64–1.43)	0.77 (0.54–1.07)	0.81 (0.64–1.03)

RTV concentrations followed a pattern similar to ATV, showing a significant drop after the addition of RIF (PK2) both in plasma and PBMC (**Table** **2**; **Figure** **S1**), even considering that PK2 concentration was evaluated at 12 hour in the PBMC compartment. In this case, a significant change in the PBMC/plasma concentration ratio was observed at PK2, from 1.69 (1.20–2.37) to 3.24 (2.42–4.42), with a GMR 1.66 (1.28–2.15). Interestingly, ATV/r dose escalation at PK3 recovered RTV trough concentrations in PBMC to levels not significantly different from PK1, with a GMR of 0.73 (0.47–1.13). Further increasing the RIF dose at PK4 resulted in a slight reduction in RTV concentration in plasma compared with PK3. Still, this difference was absent (almost reverted) in the PBMC compartment, with a GMR of 1.20 (CI_90_ 0.80–1.80). This phenomenon was associated with a significant increase in the PBMC/plasma ratio at PK4 compared with both PK3 and PK1, as reported in **Table** **3**.

### Intracellular DTG concentrations

DTG concentrations in PBMC, GMR, and the PBMC/plasma concentration ratios at the different PK visits are reported in **Tables** **2** and **3**, respectively.

Briefly, DTG concentrations increased significantly from a geometric mean of 417 ng/ml (295–661) at PK2 to 872 ng/ml (661–1,175) at PK3, with a GMR of 1.94 (1.34–2.83), in accordance with plasma concentrations. Further, increasing the RIF dose yielded a reduction limited to the PBMC compartment, with a GMR of 0.78 (0.62–0.98), while plasma concentrations showed no reduction.

### Correlations between plasma PK parameters and concentrations in PBMC

By testing the correlation of the *C*_max_, *C*_trough_, and AUC data in plasma with PBMC, we observed that ATV concentrations in PBMC at 24 hour (PK1) and 12 hour (PK2 to 4) strongly correlated with both the corresponding concentration in plasma at the same timing and with the overall exposure in the dosing interval (AUC_0‐tau_). A similar association between plasma and PBMC concentration was observed for RTV, except for PK2; nevertheless, the correlation between the concentration in PBMC and the AUC_0‐tau_ was limited to PK2 and PK4 for RTV.

Only a mild correlation (ρ < 0.5, *P* > 0.01) was observed with the *C*_max_ for ATV at PK2 to 4 when the concentration in PBMC was evaluated at 12 hour, while no association with the *C*_max_ was observed for RTV at any timepoint.

Concerning DTG, strong correlations were found between *C*_trough_ in PBMC and the corresponding concentration in plasma at PK2 and PK3, while the correlation seemed lower with the *C*_max_ in plasma and not significant (except at PK3) with the AUC_0‐tau_.

No significant correlation was observed at PK4.

### Associations with safety data

No serious adverse events were reported during the trial, with only one grade 1 ALT elevation after the addition of RIF (period 2, before PK2) and 2 after ATV/r dose escalation (period 3, before PK3). No rebound in viremia was observed in any participant. Testing correlations of ATV and RTV concentrations in plasma and PBMC with ALT, AST, GGT and bilirubin, no significant correlations emerged between these variables or with their changes during the protocol, compared with the baseline.

## DISCUSSION

The DERIVE study showed that the escalation of ATV/r dose from 300/100 mg once to twice daily is sufficient to overcome the DDI with RIF considering the theoretical PK/PD target concentrations in most participants. We have now confirmed the evidence generated from the PK in plasma at the intracellular level, showing comparable trough concentrations in PBMC with the standard 300/100 q.d. dosing (PK1) and in the presence of RIF, after ATV/r dose escalation (PK3). This confirmation at the intracellular level is very important since the intracellular compartmentalization of P‐gP substrates, such as ATV, RTV, and DTG, can be affected by its expression and activity in PBMC, which is induced by RIF, but inhibited by ATV/r^5^, ^10^, ^12^, ^29^, ^36^: this means that sufficient plasma concentrations after dose escalation could correspond to lower (and possibly insufficient) exposure in PBMC.

Interestingly, no significant difference was observed between ATV or RTV concentrations in PBMC after the further increase in RIF dose (PK3 vs. PK4), confirming that the inductive effect of RIF on CYP3A reaches a ceiling over 600 mg/day, as previously shown by Lutz *et al*.,^37^, ^38^ and that the increase in its dose does not significantly affect the intracellular partitioning of ATV and RTV. This is particularly important from a clinical point of view since some previous works evidenced a faster response to the antitubercular treatment in the presence of higher doses of RIF (1,200 mg/day)^39^ and a relatively high prevalence of suboptimal RIF concentrations with standard dosing.^40^ All these aspects are particularly clinically relevant in sub‐Saharan Africa, where the prevalence of HIV‐TB coinfection is high, since this allows clinicians to start first‐line anti‐TB treatment with RIF in PLWH treated with ATV/r by simply doubling their dosing schedule to twice daily, even in case of use of high dose RIF. This avoids tricky therapeutic changes, with the possible emergence of new adverse events or lack of virological control, while maintaining pharmacoeconomic sustainability.

The results from this study reported both intracellular concentration data and the PBMC/plasma concentration ratios for each drug, together with the respective GMR. These parameters served different scopes. First, the intracellular concentrations data and their GMR allowed us to verify if significant underexposure in PBMC, was compared with the baseline, and were further compared with the literature reported PA‐IC_90_, as cautionary reference levels. It is worth noting that all the patients had controlled viremia at baseline so the observed intracellular concentrations were surely active. On the other hand, the relative penetration ratios and their GMR allowed us to assess if the eventual changes in the intracellular concentrations were explained by the same changes in plasma or specific modifications in the compartmentalization process, such as differences in the transport. Regarding ATV, the evidence of similar GMR in PBMC and plasma concentrations between PK3 and PK1, combined with not significant increase in the penetration ratio, suggests that the recovery of the intracellular trough concentration is prevalently sustained by the recovery in plasma, due to compensation of the increased clearance by the reduced dosing interval and additional enzymes/transporters inhibition by the increased ATV/r dose, as well modeled by Montanha *et al*., with a possible minimal contribution by increased compartmentalization and/or delayed equilibration of intracellular concentrations relative to plasma.

The strength of our study is the prevalence of females and black Africans among the volunteers, a population well representing the target patients, while most trials are conducted in Caucasian males. Compared with recent literature data about intracellular ATV concentrations in women, we observed lower PBMC/plasma ratios for ATV with the standard regimen, at PK1 (C12 hour), while these ratios became more consistent with previous reports at the following timepoint (C12 hour).^41^ This relative change in the PBMC/plasma ratios, although not significant, could suggest some differences in the compartmentalization of ATV depending on the timing of blood withdrawal. These differences reached statistical significance for RTV, which resulted in more partitioned in PBMC, relative to plasma, at PK2 and PK4, when the lowest concentrations were found in plasma. This suggests a buffering effect on the concentrations in the intracellular compartment, maybe due to slightly delayed equilibration of intracellular concentrations relative to plasma, as previously described.^42^ On the other hand, the PBMC/plasma ratio for DTG at all the time points was comparable to what was described in previous works.^24^, ^33^, ^34^

Concerning DTG trough concentrations in PBMC, a significant increase was observed after ATV/r dose escalation, highlighting the capability of ATV/r to increase DTG exposure, even at the intracellular level. It is important to note that the increase was quite proportional between PBMC and plasma concentrations at PK3, as indicated by the GMR of PBMC/plasma concentration ratios, which was nearly 1. Conversely, the intracellular partitioning seemed to be slightly reduced in the presence of high dose RIF, suggesting that some further induction of drug transporters on the PBMC membrane, had a prevalent effect on DTG (already characterized by a relatively low intracellular penetration), compared with ATV and RTV.^36^

Concerning the PK/PD implications of the observed data, we cannot make a clear inference about the antiviral efficacy of the drug concentrations in PBMC, due to the absence of generally acknowledged and appropriate concentration cutoff values which are predictive of viral control in the cellular compartment. In this work, we used as a general reference the values of PA‐IC_90_ for ATV and DTG, as suggested in some previous works.^43^ Nevertheless, this represents a surrogate, conservative and precautionary measure since the intracellular drug concentrations are not expected to be bound to intracellular proteins or cellular organelles to the same extent as to plasma proteins. Therefore, the intracellular concentration is expected to be more “active” than the one observed in plasma. Using these surrogate reference values, we observed encouraging results for ATV, reporting concentrations well above the PA‐IC_90_ of 14 ng/ml after dose escalation (PK3 and PK4), further confirming the theoretical virological safety of this strategy.

Nevertheless, it is important to remember that the PA‐IC_90_ value of 14 ng/ml is appropriate for wild‐type viruses and may be suboptimal for some viral strains with reduced susceptibility to PIs, which may need increased drug exposure.

On the other hand, the observed increased plasma and intracellular exposure for DTG to values well higher than the ones expected with q.d. dose without RIF and ATV/r,^33^, ^44^ opens the way to some hypotheses, particularly considering recent literature. In fact, a recent study showed that the standard DTG dose of 50 mg/day is sufficient to maintain viral control during antitubercular treatment with a standard dose RIF.^45^ Merging this evidence, in the presence of ATV/r, once‐daily DTG may be robust even in the presence of high dose RIF (1,200 mg/day), since ATV/r would work as a PK booster for DTG, counterbalancing the effect of RIF. Nevertheless, further investigation is needed to confirm this. On the other, the evidence of a slight reduction in the intracellular DTG concentration with high RIF dose (between 2 and 38%) is not expected to be clinically significant in the case of twice daily DTG, since expected concentrations in plasma would still exceed the PA‐IC_90_ by far,^45^ but it may be potentially concerning in case of once daily use with concomitant high dose RIF, without the boosting effect of ATV/r, and this may deserve investigation in the near future.

A previous study reported higher recovery of ARVs with a “spin through oil” approach using Nyosil‐M25 oil, compared with classical washing methods, implying that these could underestimate the intracellular penetration.^46^ Despite this, in this study, we applied the PBMC isolation methodology based on two fast cell washing steps with cold isotonic solution for the following reasons: this protocol eliminates the influence of extracellular drug concentrations and provided, in preliminary experiments, contained changes in drug concentrations (mean 10% for ATV and 18% for DTG) compared with one single washing; the comparative washing steps investigated by Cory *et al*. took longer than those used in the present study, so the drug loss in this work is expected to be more contained; this same isolation protocol was used in previous works obtaining comparable results for intracellular DTG and other drugs with studies which used the spin through oil approach^24^; ARVs can partition to oil and some oil residues within cell pellets could cause overestimation of drugs concentrations and/or progressive contamination of mass spectrometric detectors,^47^ and finally, a possible slight constant underestimation of intracellular concentrations is not impacting the GMR between treatment periods, thus not impacting in a relevant manner on the pharmacological and clinical relevance of this study, as already reported in some previous works.^34^

The present study has some limitations. The quantification of ARVs in PBMCs was performed just at 24 or 12 hours, to limit blood withdrawal, but this only allows observation of intracellular *C*_trough_ at PK1, 3, and 4, while the comparison between the *C*_trough_ at PK1 and the C12 hour at PK2 clearly underestimates the drop in intracellular concentrations after the introduction of RIF (since at this timing ATV/r was given once daily). This means that as expected, the actual trough concentration at PK2 would probably not be quantifiable in several cases.

Another limitation is the current inability to understand the exact disposition of intra‐PBMC concentrations within the cell. The targets of the drugs are situated in different places within the cell: ATV/r targets are within the cytoplasm, while the molecular target of DTG is within the nucleus. This implies that, despite the clear advantage that quantification in PBMC is more informative and targeted to the right cell types, some caution is still needed when considering intracellular concentration from a PK/PD perspective. Additionally, this study involved healthy PLWH, without TB infection and taking only RIF, so these results do not consider the possible effect of inflammation on the expression of drug CYPs and transporters,^48^ nor CYP inhibition by concomitant isoniazid, which is another key component of first‐line antitubercular treatment.^49^ However, in this case, a clinically relevant impact from isoniazid would probably be negligible, since its effect as an enzyme inhibitor is known to be reversible and primarily relevant at peak concentrations, too short to counterbalance enzyme induction by RIF.^49^ Finally, since the study was mainly focused on the PK DDI and DTG was added as a safety drug, together with maintenance of the NRTI backbone, we cannot make any inference about the actual virological effectiveness, since many drugs were present at theoretically active concentrations, explaining the observed control of viremia.

Notably, there was an important lack of association between drug concentrations in PBMC or plasma with the changes in transaminase levels, suggesting that the observed minor changes in ALT and AST were unrelated to the exposure to ARVs in plasma or within the cells. Nevertheless, since the drug exposure in PBMC can differ significantly from that in hepatocytes, this evidence cannot be directly translated and remains speculative.

The absence of relevant liver toxicity in this study, compared with similar works on other bPIs,^15^, ^16^, ^17^, ^18^ can be explained by different factors: the enrolled patients were already in ATV/r regimens and did not switch treatment, so they were “pre‐selected” to have a good tolerance for these drugs; the time between the introduction of RIF and ATV/r dose escalation was 2 weeks, leaving the time to the enzyme induction and liver markers to stabilize to a new equilibrium; then, excluding the “Gilbert‐like” effect increasing effect of ATV on unconjugated bilirubin, due to its capability to inhibit UGT1A1, it is not expected to yield significant increase in liver enzymes^50^ even in association with RIF^14^; Associated with ATV, we observed lower intracellular concentrations of RTV than in a previous work, where RTV was associated with DRV and this could cause reduced production of reactive metabolites of RTV (M1 and M13) by CYP3A4,^4^, ^34^ which were described as determinants of liver toxicity of boosted PIs with RIF.

## CONCLUSION

In this study, we demonstrated that dose escalation of ATV/r 300/100 mg from once to twice daily in the presence of RIF restored ATV concentrations within the cell comparable to those prior to the addition of RIF. The evidence of sufficient intracellular exposure to ATV, together with the encouraging tolerability data, gives more confidence to advance this approach to future clinical practice in countries with high incidences of HIV and tuberculosis coinfection.

## FUNDING

This project is part of the EDCTP2 program supported by the European Union (grant number RIA2016MC‐1606‐VirTUAL).

## CONFLICT OF INTEREST

The authors declared no competing interests in this work.

## AUTHOR CONTRIBUTIONS

All the authors contributed to the research as follows: A.D.N., A.P., P.D., and C.W. wrote the manuscript. A.D.N., H.M., C.W., A.D., S.K., G.M., and H.M. designed the research. A.D.N., A.P., H.M., S.N., J.N., J.K., D.O., M.L., K.G., and L.W. performed the research. A.D.N., A.K., and K.G. analyzed the data.

## Supporting information


Figure S1.
