# Supplementary material for: Intracellular Penetration of Atazanavir, Ritonavir and Dolutegravir With Concomitant Rifampicin: A Dose Escalation Study
Source: Clin Pharmacol Ther. 2025 Jan 31;117(5):1393–402. doi: 10.1002/cpt.3572 (PMC11993292; doi:10.1002/cpt.3572)

Plasma PK summary

As described previously, the addition of standard dose RIF reduced the exposure to ATV at PK2: GMR of AUC and C_{trough} were 0.15 (0.12 – 0.19) and 0.039 (0.032 – 0.048), respectively [18]. ATV/RTV dose escalation to twice daily (PK3) resulted in comparable exposure to PK1, both in terms of AUC and C_{trough}, with GMR of 1.0 (0.94 – 1.2) and 0.83 (0.64 – 1.1), respectively. Finally, a further increase in RIF dose to 1200 mg did not yield a significant change in the overall exposure to ATV compared to PK 3, with GMR 0.93 (0.83 – 1.0) for the AUC and 0.98 (0.81 – 1.2) for the C_{trough}, respectively.

Another finding was, as expected, that ATV/RTV dose escalation at PK3 significantly increased the exposure to DTG twice daily compared with PK2, both in terms of AUC, with a GMR of 1.6 (1.4 – 1.7) and C_{trough} with a GMR 2.0 (1.7 – 2.3). Again, further increasing the RIF dose to 1200 mg at PK4 did not yield a significant impact on DTG exposure compared to PK3, with a GMR 0.95 (0.89 – 1.1) for the AUC_{0-24} and 0.96 (0.85 – 1.1) for the C_{trough}, respectively. RTV PK in plasma showed a similar trend compared with that of ATV.

Detailed data are summarized, together with intra-PBMC concentrations, in Table 2.

Supplementary Figure S1: Depiction of the individual trends in intra-PBMC concentrations of Ritonavir (panel A) and 90% CI of the geomean concentration (panel B) changes throughout the study. Dotted lines represent the boundaries of the CI, solid line the geomean. C12h was evaluated at PK2. PK1 = ATV/r 300/100 mg qd; PK2 = addition of RIF 600 mg qd and DTG 50 mg bid; PK3 = ATV/r dose escalation to 300/100 mg bid; PK4 = RIF dose increased to 1200 qd.

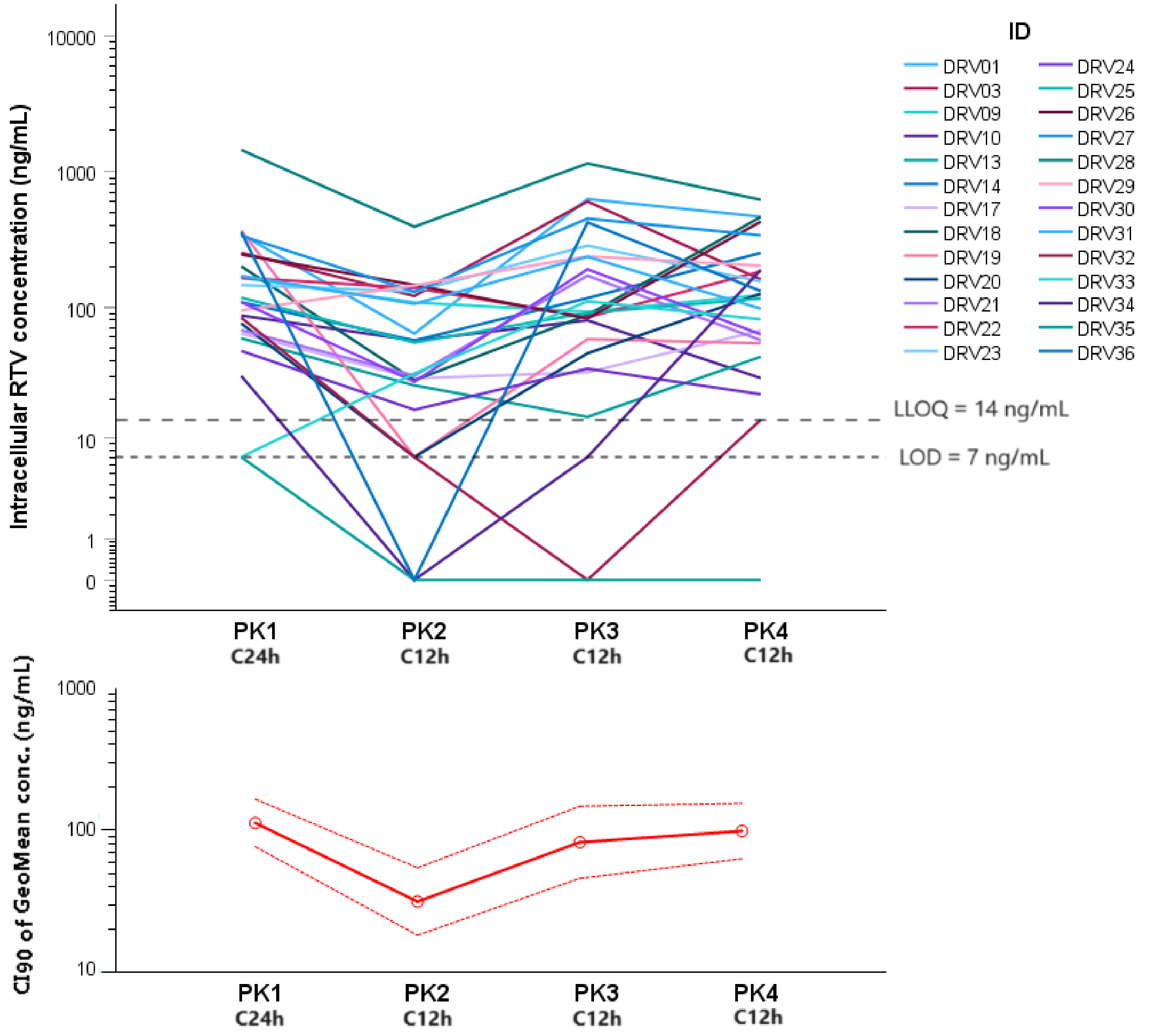

Supplement: Supplementary file 1 — Figure S1. [file CPT-117-1393-s001.pdf]
